# Monitoring of anti-drug antibodies and disease-specific biomarkers in three patients from a Japanese Fabry family treated with enzyme replacement therapy

**DOI:** 10.1007/s13730-022-00738-7

**Published:** 2022-10-07

**Authors:** Takao Kubota, Takahiro Tsukimura, Tomoko Shiga, Tadayasu Togawa, Hitoshi Sakuraba

**Affiliations:** 1 Department of Nephrology, Tohto Sangenjaya Clinic, 2-13-2 Taishido, Setagaya, Tokyo, 154-0004 Japan; 2 Department of Nephrology, Self-Defence Forces Central Hospital, 1-2-24 Ikejiri, Setagaya, Tokyo, 154-8532 Japan; 3 Department of Functional Bioanalysis, Meiji Pharmaceutical University, 2-522-1 Noshio, Kiyose, Tokyo, 204-8588 Japan; 4 Department of Clinical Genetics, Meiji Pharmaceutical University, 2-522-1 Noshio, Kiyose, Tokyo, 204-8588 Japan

**Keywords:** Fabry disease, Enzyme replacement therapy, Anti-drug antibody, Biomarker, Globotriaosylceramide, Globotriaosylsphingosine

## Abstract

We monitored anti-drug antibodies and disease-specific biomarkers in three patients with a nonsense mutation from a Japanese Fabry family treated with enzyme replacement therapy (ERT). In two male patients from the family, neutralizing anti-drug antibodies were induced at an early stage of ERT, the antibody titer peak being found at an earlier stage of ERT in the patient treated with 1.0 mg/kg agalsidase beta than in that treated with 0.2 mg/kg agalsidase alfa. Then, the antibody titers decreased with continuation of ERT. The formation of neutralizing anti-drug antibodies adversely affected the plasma globotriaosylsphingosine (Lyso-Gb3) level and urinary globotriaosylceramide (Gb3) excretion in both patients, the impact being greater in the patient treated with 0.2 mg/kg agalsidase alfa than in that treated with 1.0 mg/kg agalsidase beta. The difference might be explained by the different doses of the infused enzymes based on supersaturation of the antibodies. In a heterozygous Fabry female from the family, no sign of antibody formation was found, and both the plasma Lyso-Gb3 level and urinary Gb3 excretion, which were moderately increased at the baseline, decreased gradually. No deterioration of the manifestations or laboratory findings was observed during ERT in either of the patients. Thus, monitoring of anti-drug antibodies and biomarkers in these Fabry patients provided us with important information on their pathological condition during ERT.

## Introduction

Fabry disease is an X-linked genetic disorder caused by mutations in the *GLA* gene resulting in a defect of α-galactosidase A (α-Gal) [1]. The enzyme defect causes accumulation of glycolipids, predominantly globotriaosylceramide (Gb3) and globotriaosylsphingosine (Lyso-Gb3), in the organs/tissues and body fluids of Fabry patients. Classically affected Fabry males with deficient α-Gal activity exhibit systemic manifestations that begin in childhood or adolescence, although there are atypical Fabry males with residual α-Gal activity who exhibit milder clinical manifestations with late-onset. On the other hand, heterozygous Fabry females exhibit a more variable clinical picture, from asymptomatic to severe, due to random X-chromosomal inactivation.

As to a disease-specific therapy for Fabry disease, enzyme replacement therapy (ERT) with recombinant α-Gals (agalsidase alfa produced in human fibroblasts and agalsidase beta expressed in Chinese hamster ovary cells) has been successfully used [2, 3]. However, recent reports demonstrated that ERT often induces the formation of anti-drug antibodies, especially in classic Fabry males, which may impair the efficacy and safety of ERT in some patients [4]. Thus, the importance of detection of anti-drug antibodies and measurement of biomarkers is increasing for obtaining information on the pathologic condition of Fabry patients during ERT.

In this study, we monitored anti-drug antibodies and disease-specific biomarkers in three patients from a Japanese Fabry family treated with ERT. Of the patients analyzed, the diagnosis process in the proband (a 62-year-old female, Case 1), who had a history of palpitation, was reported previously [5]. Briefly, echocardiography and cardiac magnetic resonance demonstrated that she had marked left ventricular hypertrophy (LVH) and a localized thinned basal posterior ventricular wall. An endomyocardial biopsy followed by light microscopy showed vacuolated cardiomyocytes and electron microscopy revealed numerous lamellar inclusions. *GLA* gene analysis revealed that she was heterozygous for a nonsense mutation (*c.772G* > *T*, *p.G258X*).

## Case presentation

### Case 1

In this study, further medical evaluation was performed for Case 1, which revealed proteinuria, mulberry cells, and a moderately elevated serum creatinine level of 0.91 mg/dL (reference range: 0.4–0.8 mg/dL). A renal biopsy followed by light microscopy (38 glomeruli were observed, one of which exhibited sclerotic changes) showed foamy changes of swollen podocytes and proximal and distal tubular cells. Electron microscopy revealed myelin-like structures in these cells. Leukocyte α-Gal activity had decreased to 2 nmol/h/mg protein (controls: 17–65 nmol/h/mg protein).

**Fig. 1:**
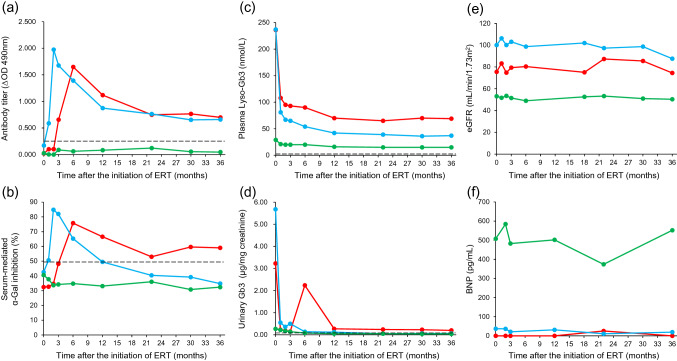
Time courses of the anti-α-Gal antibody titer (**a**), serum-mediated α-Gal inhibition rate (**b**), plasma Lyso-Gb3 level (**c**), urinary Gb3 excretion (**d**), eGFR level (**e**), and BNP level (**f**) in the Fabry patients following ERT. Green line, Case 1; red line, Case 2; and blue line, Case 3. A dotted line indicates the cut-off value

The patient was treated with 1.0 mg/kg body weight agalsidase beta biweekly, and monitoring of the anti-drug antibody titer, serum-mediated α-Gal inhibition rate, plasma Lyso-Gb3 concentration, and urinary Gb3 excretion was performed according to the methods previously described [6–8]. No increase in the anti-drug antibody titer (Fig. 1a, green line; the cut-off value indicating antibody-positive: ∆OD 490 nm > 0.250 [6]) or serum-mediated inhibition rate (Fig. 1b, green line; the cut-off value indicating inhibition-positive: > 49% [6]) was found in the patient during ERT. The plasma Lyso-Gb3 level, being moderately high at the baseline (29 nmol/L; reference range: 0.35–0.71 nmol/L [7]), decreased gradually following ERT, although it did not reach the cut-off value (Fig. 1c, green line). Urinary Gb3 excretion, being moderately increased at the baseline (0.27 μg/mg creatinine, cut-off value indicating urinary Gb3-positive: > 0.1 μg/mg creatinine [8]), decreased gradually with time (Fig. 1d, green line).

No aggravation of the symptoms or deterioration of the routine laboratory data following ERT was observed compared with at the baseline. Although a moderately low estimated glomerular filtration rate (eGFR) level (53 mL/min/1.73 m^2^) and a high brain natriuretic hormone (BNP) one (508 pg/mL) were found at the baseline, no data suggesting progression of the renal disorder and heart failure were observed during ERT (Fig. 1e, f, green line).

She had four sons, of which the eldest (Case 2) and third (Case 3) developed Fabry disease, as described below. The asymptomatic second and fourth sons were confirmed not to have Fabry disease by means of α-Gal assays.

### Case 2

A 34-year-old male, the eldest son of the proband, had a medical history of severe acroparesthesia in childhood (onset: three-year-old). Physical examination revealed hypohidrosis and sporadic angiokeratomas on his trunk. There were no abnormal findings as to blood counts and chemical tests. Urinalysis revealed proteinuria and mulberry cells. There were no abnormalities in eye and ear examinations. Cardiac assessment revealed that he had LVH. A kidney biopsy followed by pathological examination (30 glomeruli were observed, three of which exhibited sclerotic changes) showed foamy changes in swollen podocytes, and proximal and distal tubular cells, and lamellar inclusion bodies in these cells.

Enzyme assaying revealed deficient α-Gal activity (< 1 nmol/h/mg protein), and gene analysis identified the same nonsense mutation as that his mother harbored.

As the patient wanted to undergo treatment that required a short infusion period, he received ERT with 0.2 mg/kg body weight agalsidase alfa every 2 weeks. The anti-drug antibody titer value began to increase from 3 months after the initiation of ERT, and reached a peak (∆OD 490 nm = 1.65) at 6 months. Then, it gradually decreased, although it was still higher than the cut-off value (Fig. 1a, red line). The serum-mediated α-Gal inhibition rate value changed in step with the anti-drug antibody titer (Fig. 1b, red line). The plasma Lyso-Gb3 concentration, being apparently high (236 nmol/L) at the baseline, rapidly decreased within a month after the initiation of ERT, and then gradually decreased, although it stayed at a relatively high level (90–93 nmol/L) between 3 and 6 months. Then, the value reached a plateau after continuation of the ERT for 12 months (Fig. 1c, red line). A high level of urinary Gb3 excretion (3.23 μg/mg creatinine) was found at the baseline, which dramatically decreased in the first month and increased again between 3 and 12 months (peak: 6 months) after the initiation of ERT. Then a low level of Gb3 excretion continued (Fig. 1d, red line).

No aggravation of the symptoms or deterioration of the routine laboratory data following ERT was observed, and this continued for several months after the time of the last analysis shown in Fig. 1. His eGFR and BNP levels at the baseline were 76 mL/min/1.73 m^2^ and < 18.4 pg/mL, respectively, and no apparent decrease in the eGFR value (Fig. 1e, red line) or increase in the BNP one (Fig. 1f, red line) was found compared with those at the baseline during ERT.

### Case 3

A 31-year-old male, the third son of the proband, suffered from acroparesthesia in childhood (onset: 13-year-old). Physical and laboratory examinations revealed that he had hypohidrosis, angiokeratomas on his trunk, hearing loss, proteinuria, and mulberry bodies. An electrocardiogram revealed a short PQ time and bradycardia. A renal biopsy followed by pathological examination (39 glomeruli were observed, six of which exhibited sclerotic changes) showed foamy changes in podocytes, and proximal and distal tubular cells. Myelin-like structures were detected in these cells on electron microscopy.

His leukocyte α-Gal activity level was < 1 nmol/h/mg protein, and his genotype was the same as that of Case 2.

He received ERT with 1.0 mg/kg body weight agalsidase beta every other week. The anti-drug antibody titer quickly increased within a month, reached a peak (∆OD 490 nm = 1.98) at 2 months after the initiation of ERT, and then gradually decreased, although it did not reach the cut-off value (Fig. 1a, blue line). The serum-mediated inhibition rate value changed in step with the anti-drug antibody titer and remained at a value less than the cut-off one from 12 months after the initiation of ERT (Fig. 1b, blue line). The plasma Lyso-Gb3 level, being apparently high (237 nmol/L) at the baseline, quickly decreased within a month, and then gradually decreased with a short period of stagnation at a relatively high level (65–67 nmol/L) between 2 and 3 months after the initiation of ERT (Fig. 1c, blue line). The urinary Gb3 excretion level, being apparently high (5.68 μg/mg creatinine) at the baseline, dramatically decreased within 1–2 months, and then gradually decreased to around the cut-off value, although a small peak was found at 3 months after the initiation of ERT (Fig. 1d, blue line).

No aggravation of the symptoms or deterioration of the routine laboratory data following ERT was found in the patient, as in Case 2. His eGFR and BNP levels at the baseline were 100 mL/min/1.73 m^2^ and 37 pg/mL, respectively, and there were no findings that suggested progression of the renal dysfunction or cardiac failure (Fig. 1e, f, blue line).

## Discussion

An intensive search for biomarkers useful for supporting a diagnosis and monitoring of therapy for Fabry disease has been conducted [9], and Gb3 and Lyso-Gb3, which accumulate in the organs/tissues and body fluids of Fabry patients, have attracted attention as available specific biomarkers for the disease. Many investigators have reported that the plasma Lyso-Gb3 and urinary Gb3 levels in classic Fabry males are high at the baseline and that they decrease with ERT [7, 10, 11]. The accumulation of Gb3 in all renal cell types, including podocytes, endothelial cells, epithelial cells, and tubular cells, possibly occurs in Fabry patients, which would contribute to the renal involvement [1, 12]. Nephropathy is one of the main disorders, determining the prognosis of this disease, and urinary Gb3 excretion is expected to be a biomarker associated with renal involvement.

In this study, we monitored the plasma Lyso-Gb3 level and urinary Gb3 excretion, in addition to anti-drug antibodies, in three patients from a Fabry family with a nonsense mutation during ERT.

In Case 1, who exhibited residual α-Gal activity, no signs suggesting the formation of anti-drug antibodies were observed during ERT. Her plasma Lyso-Gb3 and urinary Gb3 levels, being moderately increased at the baseline, gradually decreased following ERT. Although she developed renal and cardiac involvement at the baseline, no apparent worsening of symptoms or deterioration of laboratory findings was found after the initiation of ERT, at least for 3 years.

On the other hand, increases in the anti-drug antibody titer and serum-mediated α-Gal inhibition rate were observed in Cases 2 and 3 at an early stage of ERT. Both the appearance of antibodies and an antibody titer peak were found at an earlier stage of ERT in Case 3 treated with 1.0 mg/kg agalsidase beta than in Case 2 treated with 0.2 mg/kg agalsidase alfa. Then, the antibody titers gradually decreased. A systemic review revealed that the incidence of anti-drug antibody formation reached 20.0–56.0% of Fabry males treated with 0.2 mg/kg agalsidase alfa and 72.7–91.1% of those treated with 1.0 mg/kg agalsidase beta and that the antibodies developed during the first 3 months of ERT in most of the male patients [4]. Furthermore, it has been reported that tolerance develops during continuing ERT in some Fabry patients whose antibody titers gradually decrease, although the high antibody titer is maintained for a long time in others [6]. Considering the time courses of the plasma Lyso-Gb3 level and urinary Gb3 excretion, the formation of neutralizing anti-drug antibodies adversely affected the levels of the biomarkers in Cases 2 and 3. Lenders et al. reported that agalsidase inhibition was associated with higher Lyso-Gb3 levels and worse disease severity scores [13]. They also reported that agalsidase ERT can dose-dependently saturate anti-drug antibody-binding sites during infusion, and that agalsidase alfa and agalsidase beta exhibit similar antibody-binding capacities and comparable saturation frequencies. They described that saturated patients (who had excess agalsidase after infusion) experienced better outcomes and biochemical responses compared with non-saturated ones, although dose escalation could lead to increased antibody titers overtime in some other patients [14]. Rombach et al. reported that the decline of plasma Lyso-Gb3 in the anti-drug antibody positive group treated with 0.2 mg/kg agalsidase alfa or beta was less pronounced than in the anti-drug antibody positive group treated with 1.0 mg/kg agalsidase beta [15]. These data and considerations may explain the results of our analysis. Considering that Cases 2 and 3 are brothers who have the same *GLA* gene mutation and developed the similar clinical symptoms, the ERT doses infused possibly influenced their anti-drug antibody formation and supersaturation of the antibodies.

A review reported that neutralizing anti-drug antibodies directly inactivate the recombinant enzymes infused and/or form antibody-enzyme complexes, leading to a decrease in the cellular uptake of active enzymes, and that the results of animal experiments revealed the effectiveness of methotrexate and belimumab as immunosuppressive drugs [16]. However, as far as we know, protocols to prevent ERT-naïve Fabry patients from forming neutralizing anti-drug antibodies have not been established yet.

In conclusion, monitoring of anti-drug antibodies and disease-specific biomarkers in patients from a Fabry family gave us important information on their pathological condition during ERT, which was useful for treatment management.
